# 5-Hy­droxy­indan-1-one

**DOI:** 10.1107/S1600536811010956

**Published:** 2011-03-31

**Authors:** Kew-Yu Chen, Tzu-Chien Fang, Ming-Jen Chang

**Affiliations:** a Department of Chemical Engineering, Feng Chia University, 40724 Taichung, Taiwan

## Abstract

In the title compound (5HIN), C_9_H_8_O_2_, is perfectly planar as all atoms, except the H atoms of both CH_2_ groups, lie on a crystallographic mirror plane. In the crystal, mol­ecules are linked by strong inter­molecular O—H⋯O hydrogen bonds, forming an infinite chain along [100], generating a *C*(8) motif.

## Related literature

For the spectroscopy of the title compound, see: Magnusson *et al.* (1964 ▶). For the synthetic and biological applications on indanones, see: Cai *et al.* (2005 ▶); De Paulis *et al.* (1981 ▶); Howbert & Crowell (1990 ▶); Kwiecien *et al.* (1991 ▶). For the preparation, see: Danishefsky *et al.* (1979 ▶). For related structures, see: Chen *et al.* (2011 ▶); Li *et al.* (2007 ▶); Saeed & Bolte (2007 ▶). For graph-set theory, see: Bernstein *et al.* (1995 ▶).
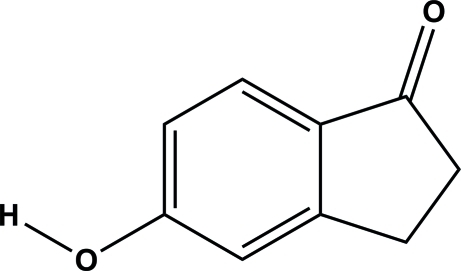


## Experimental

### 

#### Crystal data

C_9_H_8_O_2_
*M* *_r_* = 148.15
Orthorhombic, 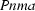

*a* = 13.9126 (7) Å
*b* = 6.7332 (4) Å
*c* = 7.5368 (3) Å
*V* = 706.02 (6) Å^3^
*Z* = 4
Mo *K*α radiation
μ = 0.10 mm^−1^
*T* = 297 K
0.39 × 0.30 × 0.25 mm

#### Data collection

Bruker SMART CCD detector diffractometer
Absorption correction: multi-scan (*SADABS*; Bruker, 2001 ▶) *T*_min_ = 0.991, *T*_max_ = 1.000
2023 measured reflections
920 independent reflections
605 reflections with *I* > 2σ(*I*)
*R*_int_ = 0.020

#### Refinement

*R*[*F* ^2^ > 2σ(*F* ^2^)] = 0.037
*wR*(*F* ^2^) = 0.081
*S* = 1.03
920 reflections
78 parameters
H atoms treated by a mixture of independent and constrained refinement
Δρ_max_ = 0.21 e Å^−3^
Δρ_min_ = −0.21 e Å^−3^

### 

Data collection: *SMART* (Bruker, 2001 ▶); cell refinement: *SAINT* (Bruker, 2001 ▶); data reduction: *SAINT*; program(s) used to solve structure: *SHELXS97* (Sheldrick, 2008 ▶); program(s) used to refine structure: *SHELXL97* (Sheldrick, 2008 ▶); molecular graphics: *ORTEP-3 for Windows* (Farrugia, 1997 ▶); software used to prepare material for publication: *WinGX* publication routines (Farrugia, 1999 ▶).

## Supplementary Material

Crystal structure: contains datablocks global, I. DOI: 10.1107/S1600536811010956/si2345sup1.cif

Structure factors: contains datablocks I. DOI: 10.1107/S1600536811010956/si2345Isup2.hkl

Additional supplementary materials:  crystallographic information; 3D view; checkCIF report

## Figures and Tables

**Table 1:** Hydrogen-bond geometry (Å, °)

*D*—H⋯*A*	*D*—H	H⋯*A*	*D*⋯*A*	*D*—H⋯*A*
O2—H2*A*⋯O1^i^	0.98 (2)	1.69 (2)	2.6618 (19)	173 (2)

Symmetry code: (i) 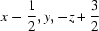.

**Fig. 1.**
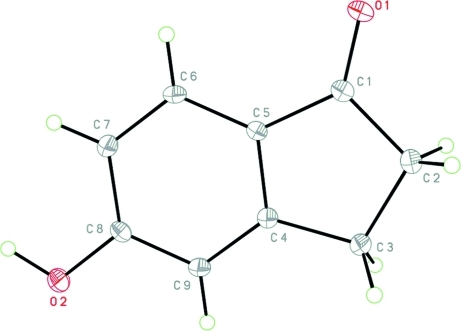
The molecular structure of the title compound, showing 50% probability displacement ellipsoids.

**Fig. 2.**
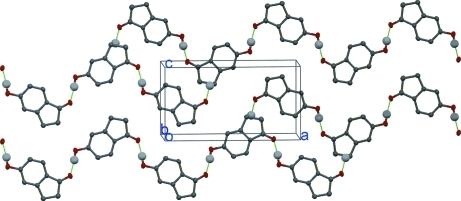
A section of the crystal packing of the title compound, viewed along the b axis. For clarity, hydrogen atoms not involved in hydrogen bonding have been omitted.

## supplementary crystallographic information

### Comment

Acid strengths of 5- and 7-hydroxyindan-1-one have been investigated by UV-vis and ^1^H NMR measurements (Magnusson *et al.*, 1964). In addition, 1-indanones were important precursors in the regiospecific synthesis of 2-fluoro-1-naphthols (Cai *et al.*, 2005). 5-Chloro-1-indanone was used to synthesize important biomedical compounds as anticonvulsants (Kwiecien *et al.*, 1991), and anticholinergics (De Paulis *et al.*, 1981), showing great activity against solid tumours (Howbert *et al.*, 1990).

The *ORTEP* diagram of the title compound (5HIN) is shown in Figure 1. The complete molecule (exceptions: H2B and H3A) is perfectly planar, which is slightly different from those of previous studies on other 1-indanone derivatives. (Chen, *et al.*, 2011; Li, *et al.*, 2007; Saeed *et al.*, 2007). In the crystal (Figure 2), the molecules are linked by strong intermolecular O—H···O hydrogen bonds (1.69 (2)Å of O2—H2A···O1 distance and 173 (2)° of O2—H2A—O1, Table 1) to form an infinite one-dimensional chain along [1 0 0], generating a C(8) motif (Bernstein *et al.*, 1995).

### Experimental

5-Hydroxyindan-1-one was purchased from Sigma-Aldrich (>95% purity) and used as received without further purification. Yellow needle-shaped crystals suitable for the crystallographic studies reported here were isolated over a period of three weeks by slow evaporation from a ethyl acetate solution.

### Refinement

H atoms bonded to O and C atoms were located in a difference electron density map. The hydroxy H atom and the C_sp3_ H atoms were freely refined, and the C_sp2_ H atoms repositioned geometrically and refined using a riding model, [C—H = 0.93 Å and *U*_iso_(H) = 1.2*U*_eq_(C)].

### Crystal data

**Table:**

C_9_H_8_O_2_	*F*(000) = 312
*M**_r_* = 148.15	*D*_x_ = 1.394 Mg m^−^^3^
Orthorhombic, *P**n**m**a*	Mo *K*α radiation, λ = 0.71073 Å
Hall symbol: -P 2ac 2n	Cell parameters from 962 reflections
*a* = 13.9126 (7) Å	θ = 2.9–29.1°
*b* = 6.7332 (4) Å	µ = 0.10 mm^−^^1^
*c* = 7.5368 (3) Å	*T* = 297 K
*V* = 706.02 (6) Å^3^	Parallelepiped, yellow
*Z* = 4	0.39 × 0.30 × 0.25 mm

### Data collection

**Table:**

Bruker SMART CCD detector diffractometer	920 independent reflections
Radiation source: fine-focus sealed tube	605 reflections with *I* > 2σ(*I*)
graphite	*R*_int_ = 0.020
ω scans	θ_max_ = 29.2°, θ_min_ = 2.9°
Absorption correction: multi-scan (*SADABS*; Bruker, 2001)	*h* = −19→19
*T*_min_ = 0.991, *T*_max_ = 1.000	*k* = −9→9
2023 measured reflections	*l* = −10→10

### Refinement

**Table:**

Refinement on *F*^2^	Primary atom site location: structure-invariant direct methods
Least-squares matrix: full	Secondary atom site location: difference Fourier map
*R*[*F*^2^ > 2σ(*F*^2^)] = 0.037	Hydrogen site location: inferred from neighbouring sites
*wR*(*F*^2^) = 0.081	H atoms treated by a mixture of independent and constrained refinement
*S* = 1.03	*w* = 1/[σ^2^(*F*_o_^2^) + (0.040*P*)^2^] where *P* = (*F*_o_^2^ + 2*F*_c_^2^)/3
920 reflections	(Δ/σ)_max_ = 0.001
78 parameters	Δρ_max_ = 0.21 e Å^−^^3^
0 restraints	Δρ_min_ = −0.21 e Å^−^^3^

### Special details

**Table:**

Geometry. All e.s.d.'s (except the e.s.d. in the dihedral angle between two l.s. planes) are estimated using the full covariance matrix. The cell e.s.d.'s are taken into account individually in the estimation of e.s.d.'s in distances, angles and torsion angles; correlations between e.s.d.'s in cell parameters are only used when they are defined by crystal symmetry. An approximate (isotropic) treatment of cell e.s.d.'s is used for estimating e.s.d.'s involving l.s. planes.
Refinement. Refinement of *F*^2^ against ALL reflections. The weighted *R*-factor *wR* and goodness of fit *S* are based on *F*^2^, conventional *R*-factors *R* are based on *F*, with *F* set to zero for negative *F*^2^. The threshold expression of *F*^2^ > σ(*F*^2^) is used only for calculating *R*-factors(gt) *etc*. and is not relevant to the choice of reflections for refinement. *R*-factors based on *F*^2^ are statistically about twice as large as those based on *F*, and *R*- factors based on ALL data will be even larger.

### Fractional atomic coordinates and isotropic or equivalent isotropic displacement parameters (Å^2^)

**Table:**

	*x*	*y*	*z*	*U*_iso_*/*U*_eq_	
O1	0.80931 (8)	0.2500	0.98929 (18)	0.0531 (4)	
O2	0.34842 (9)	0.2500	0.85642 (18)	0.0454 (4)	
H2A	0.3393 (16)	0.2500	0.728 (3)	0.078 (8)*	
C1	0.73075 (12)	0.2500	1.0631 (2)	0.0342 (4)	
C2	0.71859 (13)	0.2500	1.2617 (2)	0.0380 (5)	
H2B	0.7500 (10)	0.1348 (15)	1.3102 (18)	0.058 (4)*	
C3	0.61036 (12)	0.2500	1.2963 (2)	0.0354 (4)	
H3A	0.5894 (8)	0.1337 (17)	1.3668 (17)	0.050 (4)*	
C4	0.56635 (11)	0.2500	1.1138 (2)	0.0283 (4)	
C5	0.63619 (10)	0.2500	0.9813 (2)	0.0284 (4)	
C6	0.60937 (11)	0.2500	0.8033 (2)	0.0336 (4)	
H6A	0.6558	0.2500	0.7146	0.040*	
C7	0.51359 (12)	0.2500	0.7604 (2)	0.0343 (4)	
H7A	0.4949	0.2500	0.6419	0.041*	
C8	0.44362 (12)	0.2500	0.8945 (2)	0.0311 (4)	
C9	0.47000 (12)	0.2500	1.0715 (2)	0.0320 (4)	
H9A	0.4236	0.2500	1.1603	0.038*	

### Atomic displacement parameters (Å^2^)

**Table:**

	*U*^11^	*U*^22^	*U*^33^	*U*^12^	*U*^13^	*U*^23^
O1	0.0222 (7)	0.0992 (10)	0.0379 (7)	0.000	0.0057 (7)	0.000
O2	0.0228 (7)	0.0766 (9)	0.0369 (8)	0.000	−0.0037 (6)	0.000
C1	0.0255 (10)	0.0456 (10)	0.0315 (10)	0.000	0.0013 (8)	0.000
C2	0.0293 (11)	0.0537 (12)	0.0311 (9)	0.000	−0.0035 (8)	0.000
C3	0.0277 (10)	0.0528 (11)	0.0256 (9)	0.000	0.0005 (8)	0.000
C4	0.0254 (9)	0.0337 (9)	0.0256 (8)	0.000	0.0004 (7)	0.000
C5	0.0201 (9)	0.0386 (9)	0.0264 (9)	0.000	0.0012 (7)	0.000
C6	0.0240 (10)	0.0502 (10)	0.0264 (8)	0.000	0.0069 (8)	0.000
C7	0.0300 (10)	0.0482 (10)	0.0247 (8)	0.000	−0.0014 (8)	0.000
C8	0.0203 (9)	0.0391 (9)	0.0338 (9)	0.000	−0.0009 (8)	0.000
C9	0.0239 (10)	0.0443 (9)	0.0278 (9)	0.000	0.0054 (7)	0.000

### Geometric parameters (Å, °)

**Table:**

O1—C1	1.2264 (19)	C4—C5	1.393 (2)
O2—C8	1.355 (2)	C4—C9	1.378 (2)
O2—H2A	0.97 (3)	C5—C6	1.393 (2)
C1—C5	1.453 (2)	C6—C7	1.371 (2)
C1—C2	1.506 (2)	C6—H6A	0.9300
C2—C3	1.528 (3)	C7—C8	1.403 (2)
C2—H2B	0.963 (11)	C7—H7A	0.9300
C3—C4	1.506 (2)	C8—C9	1.384 (2)
C3—H3A	0.990 (11)	C9—H9A	0.9300
			
C8—O2—H2A	109.8 (14)	C4—C5—C1	109.13 (14)
O1—C1—C5	127.92 (15)	C6—C5—C1	130.64 (15)
O1—C1—C2	123.42 (16)	C7—C6—C5	119.18 (15)
C5—C1—C2	108.65 (15)	C7—C6—H6A	120.4
C1—C2—C3	106.29 (15)	C5—C6—H6A	120.4
C1—C2—H2B	109.1 (8)	C6—C7—C8	120.28 (16)
C3—C2—H2B	112.5 (8)	C6—C7—H7A	119.9
C4—C3—C2	104.15 (14)	C8—C7—H7A	119.9
C4—C3—H3A	111.7 (7)	O2—C8—C9	117.62 (16)
C2—C3—H3A	112.4 (7)	O2—C8—C7	121.68 (15)
C5—C4—C9	120.87 (15)	C9—C8—C7	120.70 (16)
C5—C4—C3	111.78 (14)	C8—C9—C4	118.74 (16)
C9—C4—C3	127.35 (15)	C8—C9—H9A	120.6
C4—C5—C6	120.23 (14)	C4—C9—H9A	120.6

### Hydrogen-bond geometry (Å, °)

**Table:**

*D*—H···*A*	*D*—H	H···*A*	*D*···*A*	*D*—H···*A*
O2—H2A···O1^i^	0.98 (2)	1.69 (2)	2.6618 (19)	173 (2)

Symmetry codes: (i) *x*−1/2, *y*, −*z*+3/2.
